# Epidemiology of lung cancer and approaches for its prediction: a systematic review and analysis

**DOI:** 10.1186/s40880-016-0135-x

**Published:** 2016-07-30

**Authors:** Ashutosh Kumar Dubey, Umesh Gupta, Sonal Jain

**Affiliations:** Institute of Engineering and Technology, JK Lakshmipat University, Near Mahindra SEZ, P.O. Mahapura, Ajmer Road, Jaipur, Rajasthan 302 026 India

**Keywords:** Lung cancer, Incidence and mortality rates, Data mining, Evolutionary algorithms

## Abstract

**Background:**

Owing to the use of tobacco and the consumption of alcohol and adulterated food, worldwide cancer incidence is increasing at an alarming and frightening rate. Since the last decade of the twentieth century, lung cancer has been the most common cancer type. This study aimed to determine the global status of lung cancer and to evaluate the use of computational methods in the early detection of lung cancer.

**Methods:**

We used lung cancer data from the United Kingdom (UK), the United States (US), India, and Egypt. For statistical analysis, we used incidence and mortality as well as survival rates to better understand the critical state of lung cancer.

**Results:**

In the UK and the US, we found a significant decrease in lung cancer mortalities in the period of 1990–2014, whereas, in India and Egypt, such a decrease was not much promising. Additionally, we observed that, in the UK and the US, the survival rates of women with lung cancer were higher than those of men. We observed that the data mining and evolutionary algorithms were efficient in lung cancer detection.

**Conclusions:**

Our findings provide an inclusive understanding of the incidences, mortalities, and survival rates of lung cancer in the UK, the US, India, and Egypt. The combined use of data mining and evolutionary algorithm can be efficient in lung cancer detection.

## Background

Worldwide, lung cancer is the leading cause of cancer-related death. However, according to the latest medical research reports [1–3], if the nature and symptoms of cancer are correctly identified at an early stage, it can be cured. The cancer spreads to other parts of the body through the blood and lymphatic system, which is a process called metastasis, and then quickly causes the development of secondary tumors [4]. Some high-risk factors like smoking, breathing polluted air, and living in a polluted area can negatively affect the prognosis and quality of life of lung cancer patients [1, 5]. Lung cancer can also be hereditary [2].

At the beginning of the twentieth century, the incidence of lung cancer was very low, but now its incidence is increasing rapidly [4, 6]. According to the GLOBOCAN 2012 report, there have been 1.8 million new cases (incidence) of lung cancer globally, constituting 12.9% of the total estimated cancer incidence in the year 2012 [2]. Of these cases, 58.0% are from the underdeveloped countries [2]. Hungary had the highest incidence of lung cancer (51.6%), followed by Serbia (45.6%) and Korea (44.2%) [2]. In 2012, lung cancer incidences for both men and women were highest in North America; the incidences were lowest in Africa, followed by Latin America and the Caribbean. In the same year, incidence of lung cancer in men was highest in Hungary (76.6%), followed by Armenia (72.9%) and Macedonia (44.2%) [2]. In India, the lung cancer mortality is high [7]. In 2012, the World Health Organization (WHO) reported that, worldwide, lung cancer causes 1.59 million deaths [8]. Tobacco-smokers aged above 50 years are at the highest risk for lung cancer. Presently, incidence of lung cancer is low in women, but changes in lifestyle might increase it in the future.

Based on the aforementioned data, it is clear that the worldwide incidence of lung cancer is alarming; indeed, it has become the most common and fatal type of cancer. The main objectives of this study were to assess the incidence of lung cancer and the associated mortality, and to analyze the on-going research in the field of computational methods for lung cancer detection. An in-depth analysis of the current research will be helpful in the development of new techniques to detect lung cancer at an early stage.

### Data sources and methods

For this study, we collected data on lung cancer incidence and mortality in the United Kingdom (UK), the United States (US), India, and Egypt from the following sources [3]. We have also considered data sources from France and Switzerland, as these organizations collect and publish global data.

### UK

*General Register Office for Scotland:* A repository that maintains medical statistics and records of births and deaths.

*Information Services Division (ISD), Scotland:* Part of National Services Scotland. Provides health data to all, free of charge.

*National Cancer Intelligence Network (NCIN):* Established to improve clinical outcomes, cancer care, and prevention. Since April 2013, part of Public Health England.

*Northern Ireland Cancer Registry:* Established in 1994 and located in the Centre for Public Health, Queen’s University Belfast. Maintains cancer incidence and mortality data. Funded by the Public Health Agency for Northern Ireland.

*Northern Ireland Statistics and Research Agency:* A repository that maintains medical data and social research as well as records of deaths and births.

*Office for National Statistics (ONS):* A statistical institute of the UK. Collects and publishes population, social, and economic statistics.

*United Kingdom and Ireland Association of Cancer Registries (UKIACR):* Focuses on developing cancer registration in the UK and Ireland for the purpose of studying and controlling cancer.

*Welsh Cancer Intelligence and Surveillance Unit (WCISU), Wales:* The national cancer registry of Wales. Stores and publishes data on cancer incidences in Wales.

### US

*Centers for Disease Control and Prevention (CDC):* It helps in detecting and responding to new and emerging health threats. The aim of CDC is to tackle the biggest health problems that cause disability and death.

*Surveillance, Epidemiology, and End Results (SEER) database:* An authoritative source of information on cancer incidence and survival in the US.

### India

*Indian Cancer Society (ICS):* A non-profit organization established by Dr. Darab Jehangir Jussawalla and Mr. Naval Tata for cancer awareness, detection, cure, and survival. Also gathers cancer-related incidence and mortality data from different cities in India.

*Indian Council of Medical Research (ICMR):* A council in New Delhi, India, for the preparation, collocation, and support of biomedical research. Main research focus is to control communicable diseases, cancer, cardiovascular diseases, blindness, and diabetes and to develop healthcare strategies. Launched the National Cancer Registry Program (NCRP) to collect reliable cancer data, conduct epidemiologic studies, design cancer control strategies, and organize cancer awareness programs.

*Institute of Cytology and Preventive Oncology (ICPO):* It is a leading institute under the ICMR. It provides awareness, prevention strategies, and treatments of leading cancers in India.

### Egypt

*Gharbiah Population-based Cancer Registry (GPCR):* Sponsored by the Middle East Cancer Consortium and the Egyptian Ministry of Health. Publishes annual statistics of cancer incidence and mortality as well as possible control strategies.

*International Association of Cancer Registries (IACR):* A professional society that collects cancer-related incidence, mortality, and survivorship data for a specific population group.

*National Cancer Registry Program of Egypt (NCRP):* Supported by the Egyptian Ministry of Communications and Information Technology. Collects cancer data, conducts data analysis, operates training programs, and develops cancer control strategies.

### Other countries

*World Health Organization (WHO), Switzerland:* Established in 1948 in Geneva, Switzerland. Part of the United Nations. Dedicated to all matters of global health.

*International Agency for Research on Cancer (IARC), France:* A specialized cancer research agency of the WHO. Develops and enhances cancer prevention measures, identifies malignancies at the earliest possible stage, and publishes periodic reports on cancer incidence.

*GLOBOCAN 2014, France:* A project of the IARC and the WHO. Estimates cancer incidence, mortality, and prevalence at the national level for 184 countries.

Systematic procedures and methods, surveys, and existing studies yield epidemiologic indicators that are capable of showing the process and the outcomes of a disease. Based purely on calculations and numerical information, quantitative indicators or methods can be useful. Useful quantitative indicators include incidence, prevalence, and mortality. Incidence measures new cases of lung cancer in the present population, whereas mortality is the estimate of deaths due to lung cancer in the total population [9, 10]. In this study, we used incidence and mortality to elucidate the effects of lung cancer on the population.

Incidence was calculated by using the formula as follows [2, 9, 10]:

Incidence = (LCCCP/TPRCP) × 10^N^

LCCCP = Number of new lung cancer cases in the current period

TPRCP = Number of total population at risk in the current period

N = 1, 2, 3…. [Sample population]

Mortality was calculated using the formula [2, 9, 10]:

Mortality = (DCCP/TPCP) × 10^N^

DCCP = Number of death cases in the current period

TPCP = Number of total population in the current period

N = 1, 2, 3…. [Sample population].

The current period means the years considered for the calculation of incidence and mortality.

Knowing the cancer survival rate in a given population enables researchers to estimate cancer trends and patterns as well as people’s fitness levels. Net survival shows the probability of surviving cancer without considering death from other causes. Since net survival is not influenced by other causes, it gives reliable results [11]. Two general approaches were used to estimate net survival: specific survival and relative survival. Specific survival is calculated from causes of cancer deaths [11] and is used mainly for clinical trials. According to Parkin et al. [12], sometimes the cause of death may be unavailable or unreliable; in such a case, it is not possible to correctly estimate survival. However, survival from other diseases can be helpful in finding the survival status of the patient with the disease under study by finding the differences between the other diseases and the total occurrences. It can be calculated by relative survival [12].

$${\text{Relative survival rate}}\; = \;\frac{\text{Observed survival proportion}}{\text{Expected survival proportion}}\; \times \; 100\%$$

Expected survival can be calculated by Ederer I, Edere II, and Hakulinen methods. In this study, we used net survival and relative survival rates.

## Results

### Lung cancer epidemiology in the UK

We first considered the incidence and mortality of lung cancer in the UK during the period 1975–2014. These data were based on age-adjusted or age-standardized rates. Age-adjusted rates eliminate age bias, allowing reliability when different population groups are compared. Incidence and mortality varied between populations based on age, race, sex, and demographic factors. Therefore, we compared population groups of varying ages from different countries and cities.

Table 1 shows the lung cancer incidence and mortality in the UK during the years 1975–2014 [13–19]. Incidence and mortality were based on the European age-standardized rate per 100,000 people in the UK. For men, lung cancer incidence increased in 1975–1980 and gradually decreased during 1985–2014, whereas the mortality gradually decreased during 1975–2014. For women, lung cancer incidence and mortality moderately increased during 1975–2014. Many factors, such as age, genetics, pollution and radiation levels, and lifestyle, can affect the development of lung cancer [20–22]. In the UK, smoking was the principal cause of lung cancer; 86.0% of lung cancer cases were associated with smoking [23, 24].

**Table 1:** Age-standardized rates of lung cancer incidence and mortality in the United Kingdom (1975–2014)

Year	Incidence (per 100,000 people)		Mortality (per 100,000 people)	
	Men	Women	Men	Women
1975	111.9	22.6	107.9	21.5
1980	113.2	28.1	106.6	25.6
1985	109.8	33.3	100.1	28.8
1990	86.7	34.3	87.7	30.5
1995	81.4	35.3	72.8	30.5
2000	70.5	36.5	60.5	29.8
2005	63.0	38.0	53.1	30.3
2010	59.2	40.5	48.0	31.4
2011–2014	58.2	40.8	47.3	31.1

**Fig. 1:**
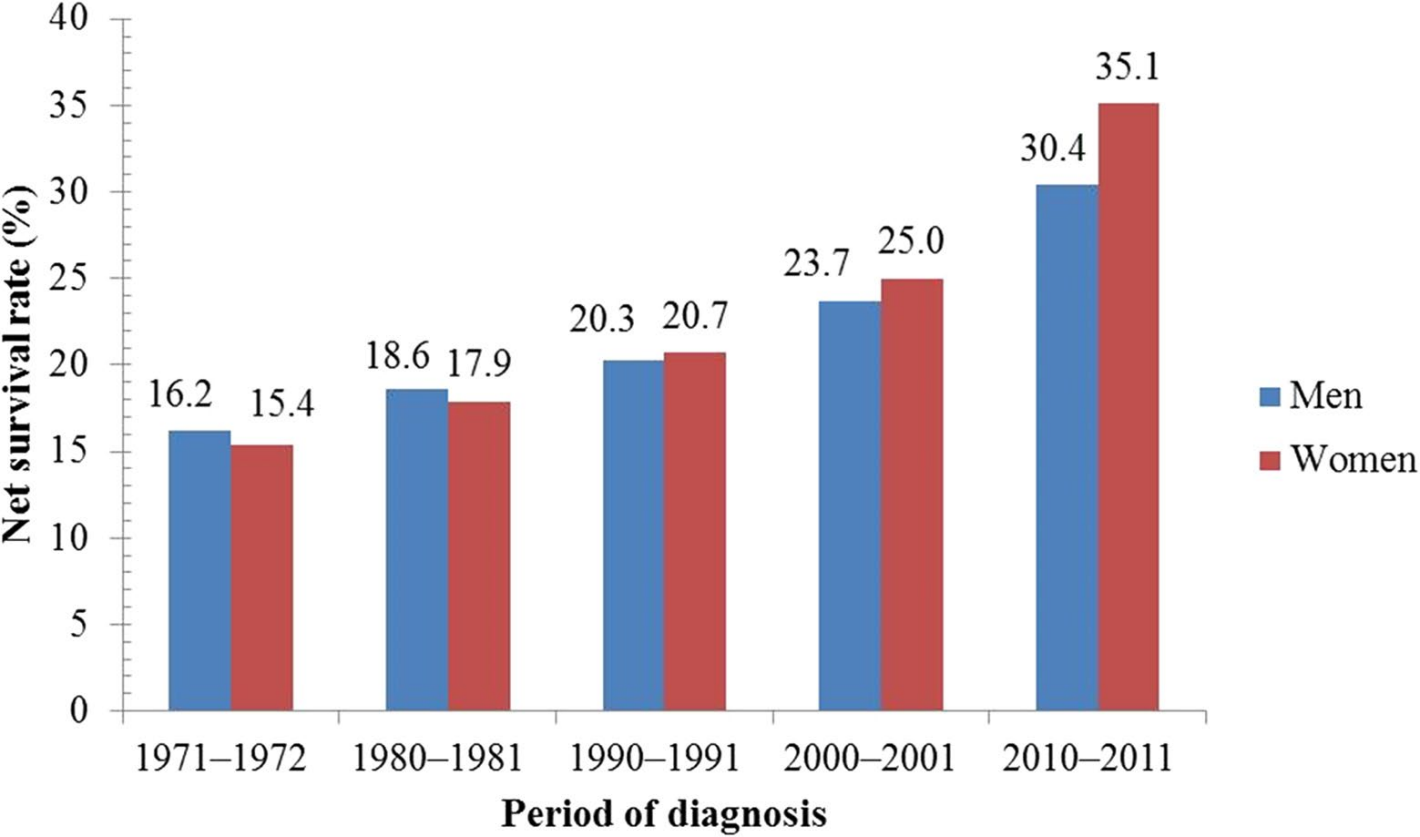
One-year net survival trends of lung cancer patients in the United Kingdom (UK). During the period 1971–2011, the 1-year age-standardized (age 15–99 years) net survival rates of men with lung cancer increased from 16.2% to 30.4%; for women, the survival rate increased from 15.4% to 35.1% during the same period

**Fig. 2:**
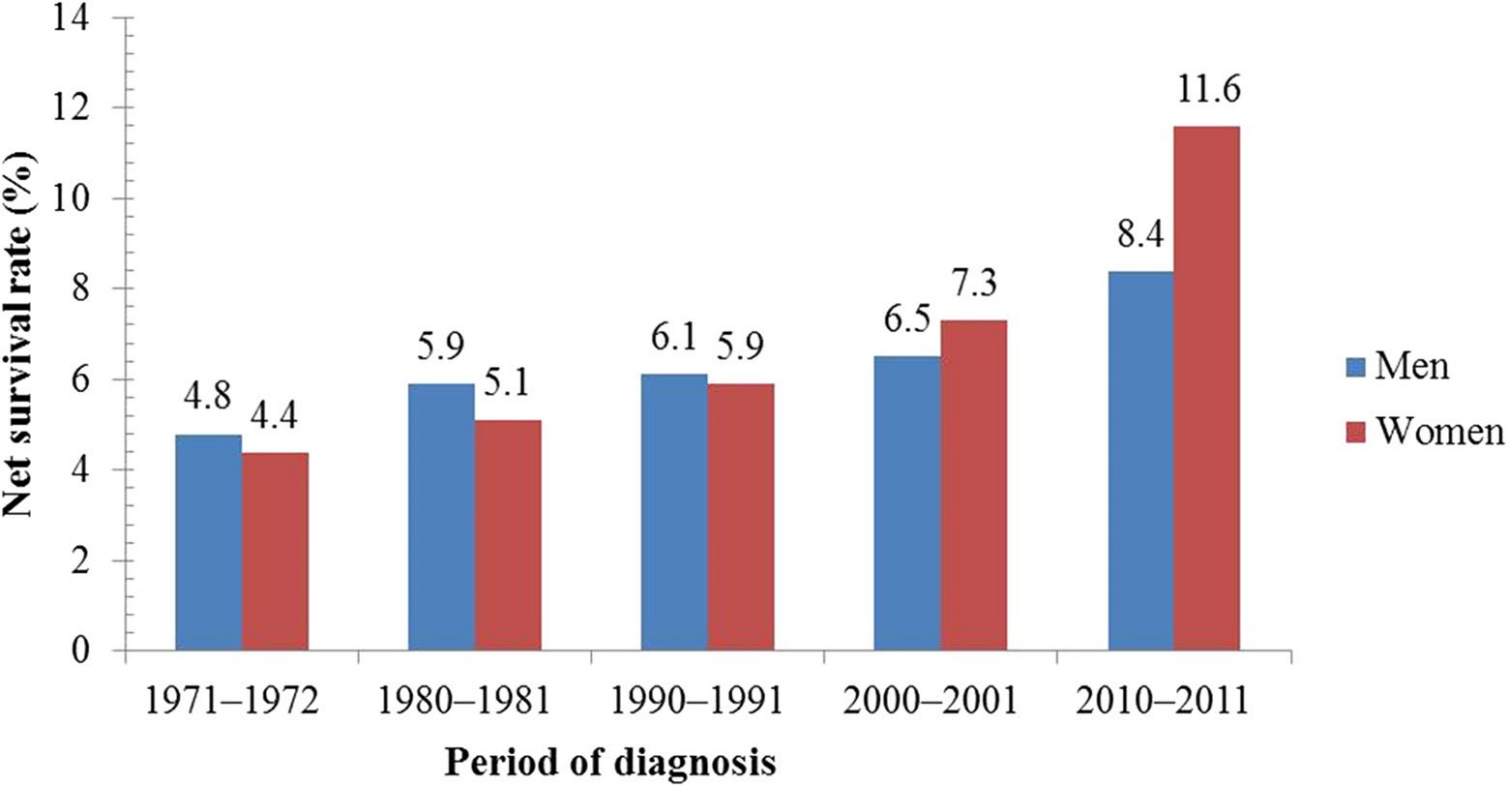
Five-year net survival trends of lung cancer patients in the UK. During the period 1971–2011, the 5-year age-standardized (age 15–99 years) net survival rates of men with lung cancer has increased from 4.8% to 8.4%; for women, the survival rate increased from 4.4% to 11.6% during the same period

**Fig. 3:**
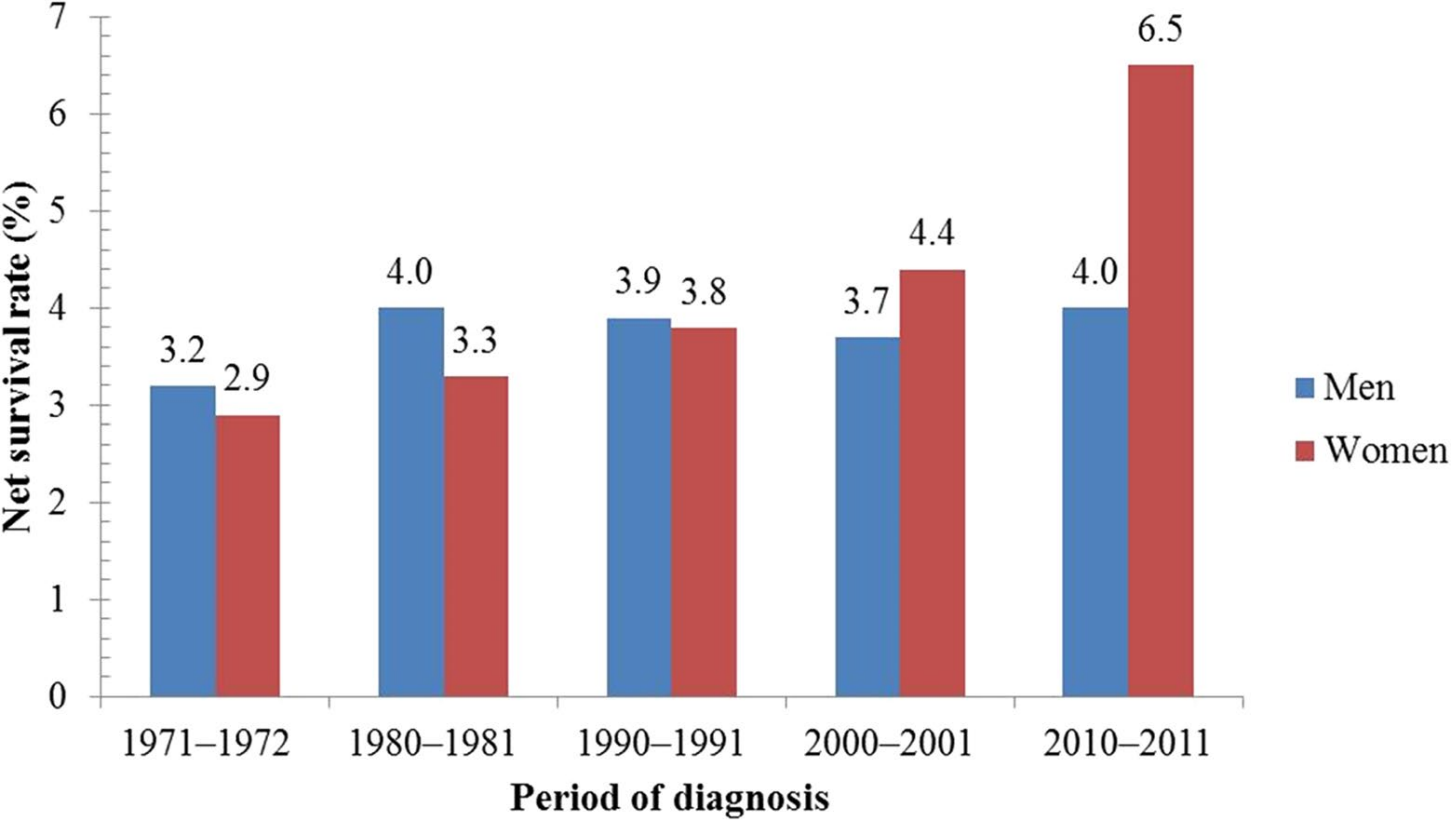
Ten-year net survival trends in the UK. During the period 1971**–**2011, the 10-year age-standardized (age 15–99 years) net survival rates of men with lung cancer increased from 3.2% to 4.0%; for women, the survival rate increased from 2.9% to 6.5% during the same period

**Fig. 4:**
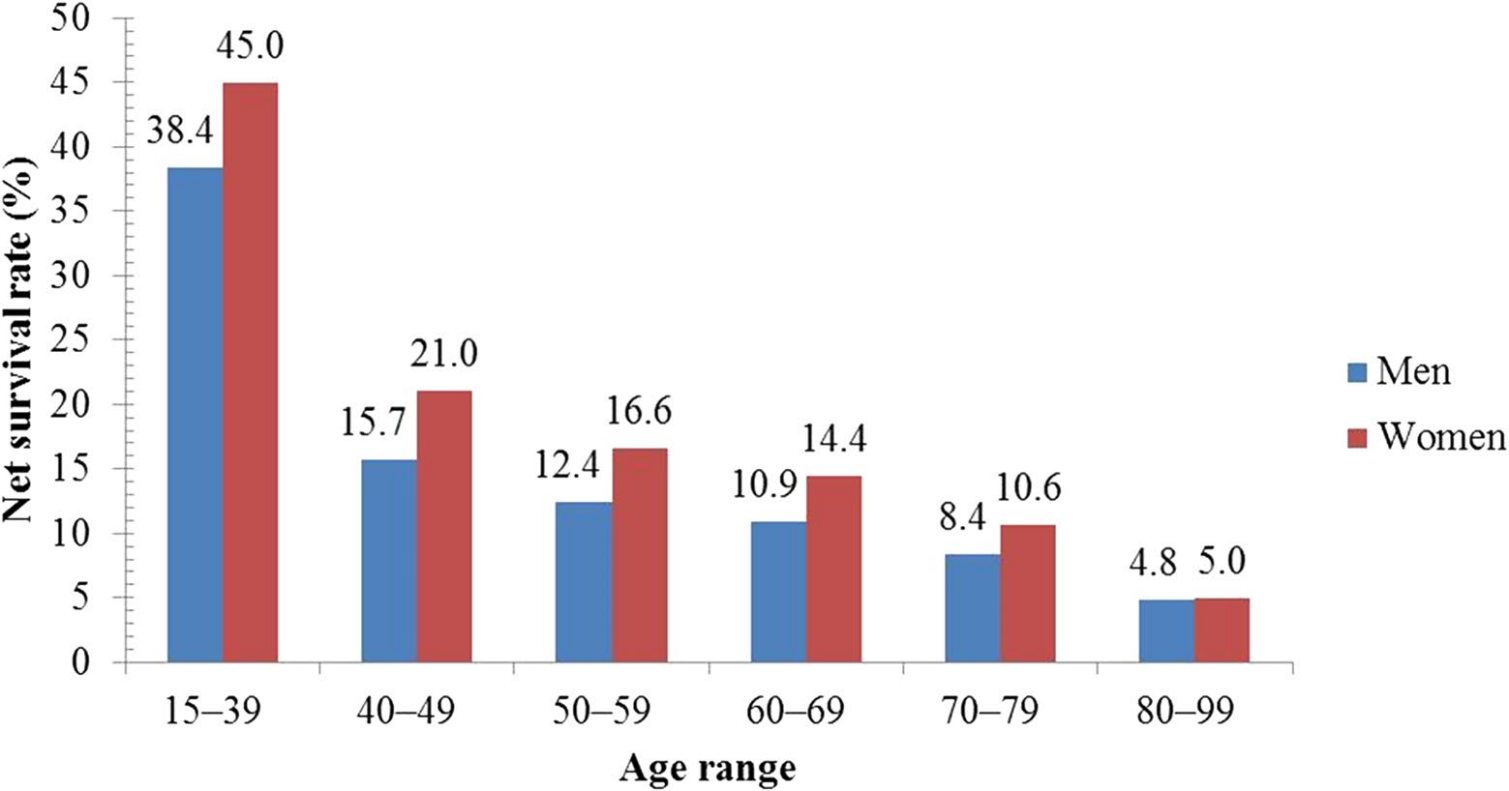
Five-year net survival rate of lung cancer patients by age in the United Kingdom (UK). During the period 2007–2011, the 5-year age-standardized net survival rates of men with lung cancer gradually decreased from 38.4% to 4.8%; for women, it decreased from 45.0% to 5.0%. This shows that the 5-year survival for lung cancer is highest in the youngest men and women and decreases with increasing age

Figures 1, 2, 3 and 4 illustrate the survival rates of men and women with lung cancer in the UK during the period 1971–2011 [13, 14, 16, 17, 25, 26]. Figure 1 shows that the 1-year survival rate increased from 16.2% to 30.4% for men and from 15.4% to 35.1% for women. Figure 2 shows that, for men, the 5-year survival rate increased from 4.8% to 8.4% in the period 1971–2011; for women, the 5-year survival rate increased from 4.4% to 11.6% in the same period. Figure 3 shows that, for men and women in the UK with lung cancer, the 10-year survival rate increased from 3.2% to 4.0% and from 2.9% to 6.5%, respectively, in the period 1971–2011. Figure 4 shows that, in the period 2007–2011, the survival rate of men and women in the UK with lung cancer gradually decreased from 38.4% to 4.8% and from 45.0% to 5.0%, respectively, with increasing age. Survival of lung cancer patients in the UK remained poor due to (i) late identification of symptoms, (ii) non-availability of optimal treatment to most patients, (iii) lack of efficient screening programs, and (iv) co-occurrence with obesity and smoking [27, 28].

### Lung cancer epidemiology in the US

Next, we examined lung cancer incidence and mortality in the US during the period 1975–2014 [29–31]. As with the data from the UK, the lung cancer data from the US were analyzed considering the age-adjusted or age-standardized rates, and SEER database incidences. For men, the lung cancer incidence increased during 1975–1980 and then gradually decreased during the period 1985–2014 (Table 2). However, mortality gradually increased during 1975–1990 and decreased moderately during 1995–2014 (Table 3). For women, the incidence of lung cancer slowly increased during 1975–2005, whereas the mortality first increased gradually during 1975–2000 and sharply thereafter.

**Table 2:** Age-adjusted rate of lung cancer incidence in the United States (1975–2014)

Year	All races (per 100,000 people)			Whites (per 100,000 people)			Blacks (per 100,000 people)		
	Both sexes	Men	Women	Both sexes	Men	Women	Both sexes	Men	Women
1975	52.2	89.5	24.5	51.9	89.1	24.8	64.5	114.9	24.7
1980	60.7	99.9	32.2	59.4	97.7	32.3	86.6	151.3	38.2
1985	64.6	98.6	40.2	63.9	96.9	40.9	89.6	149.7	46.0
1990	68.0	96.9	47.8	68.2	96.1	48.9	86.8	137.1	52.1
1995	66.8	89.8	50.4	67.1	87.9	52.4	85.8	138.7	50.0
2000	64.1	82.1	51.2	64.6	80.8	53.2	80.1	114.2	57.3
2005	62.9	75.7	53.7	63.9	75.5	55.7	74.4	97.7	59.2
2010	57.2	67.5	49.7	58.4	67.2	51.8	66.2	85.0	53.9
2011–2014	55.5	64.8	48.6	56.4	64.4	50.7	64.6	85.3	51.0

**Table 3:** Age-adjusted rate of lung cancer mortality in the United States (1975–2014)

Year	All races (per 100,000 people)			Whites (per 100,000 people)			Blacks (per 100,000 people)		
	Both sexes	Men	Women	Both sexes	Men	Women	Both sexes	Men	Women
1975	42.6	76.3	17.5	42.0	75.4	17.6	49.3	91.0	17.3
1980	49.4	84.7	24.1	48.7	83.2	24.2	59.1	106.6	24.5
1985	54.3	88.5	30.4	53.6	86.6	30.8	65.7	117.5	29.6
1990	58.8	90.5	36.8	58.1	88.4	37.3	72.0	125.1	36.4
1995	58.3	84.3	40.2	58.0	82.6	40.9	69.2	116.0	38.8
2000	55.8	76.4	41.1	55.9	75.4	42.0	63.7	100.8	39.6
2005	52.8	69.4	40.7	53.3	69.0	41.7	58.5	86.9	40.1
2010	47.4	60.0	37.9	48.1	59.9	39.1	51.1	73.5	36.2
2011–2014	46.0	57.8	37.0	46.7	57.8	38.1	49.3	70.0	35.4

**Fig. 5:**
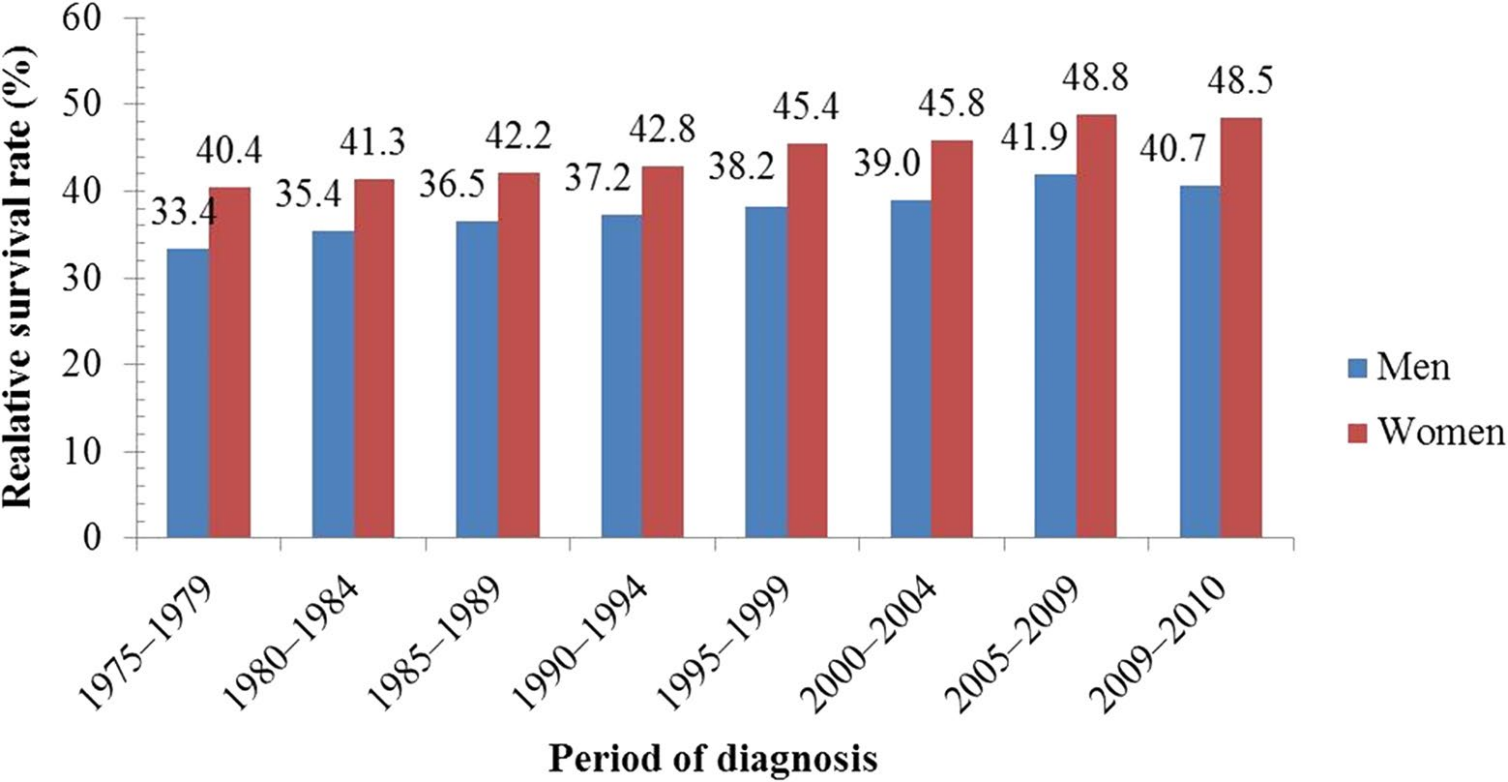
One-year relative survival trends of lung cancer patients in the United States (US). During the period 1975–2010, 1-year relative survival rate for men with lung cancer increased from 33.4% to 40.7%; for women, it increased from 40.4% to 48.5%

**Fig. 6:**
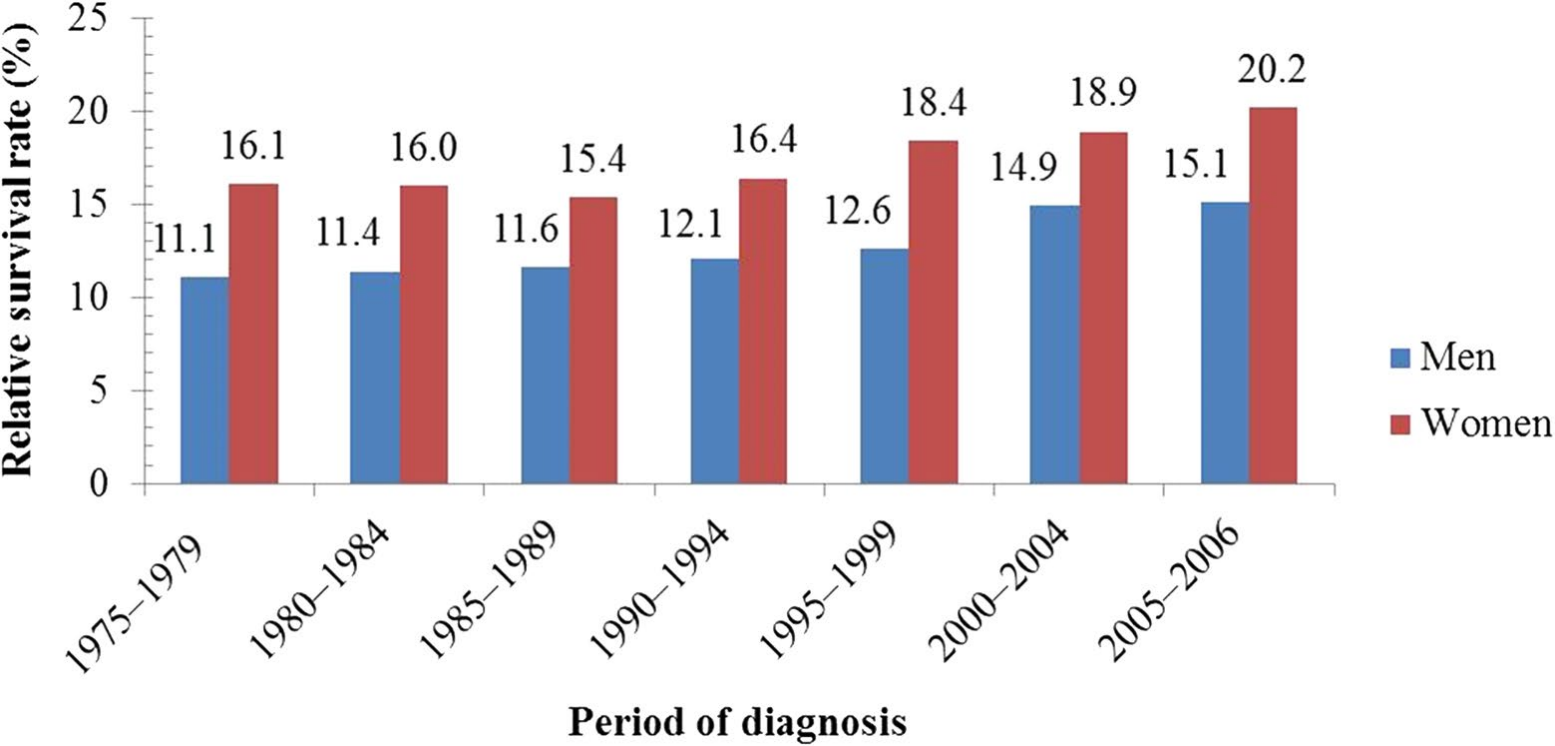
Five-year relative survival trends of lung cancer patients over time in the US. The 5-year relative survival rate of men with lung cancer increased from 11.1% to 15.1%; for women, it increased from 16.1% to 20.2%

Figure 5 shows the 1-year survival rates [32]. For men and women in the US, the 1-year lung cancer survival rate increased from 33.4% to 40.7% and from 40.4% to 48.5%, respectively, over the period 1975–2010. Figure 6 shows that in the US, the 5-year survival rate of male and female lung cancer patients increased from 11.1% to 15.1% and from 16.1% to 20.2%, respectively, during the period 1975–2006. In the year 2012, approximately 402,326 Americans had lung cancer [32]. The CDC estimated 221,220 new cases of lung cancer in 2015, representing 13.0% of all diagnosed cases of cancer [30]. Some studies showed that the chances of a person developing lung cancer depend on many factors, such as past or current smoking status, age, and sex [32]. Male smokers were at 23-times higher risk of lung cancer than male non-smokers; similarly, female smokers were at 13-times higher risk of lung cancer than female non-smokers [33].

As shown in Table 4, a 60-year-old man has a probability of 1.9% of developing lung cancer over the next 10 years; the corresponding probability for a 60-year-old woman is only 1.5% [34].

**Table 4:** Estimated probability of developing lung cancer in men and women in the United States 10, 20, and 30 years later according to their current ages (2010–2012)

Current age (years)	Lung cancer risk (%)					
	10 years		20 years		30 years	
	Men	Women	Men	Women	Men	Women
30	0.0	0.0	0.1	0.1	0.8	0.7
40	0.1	0.1	0.8	0.7	2.5	2.1
50	0.6	0.5	2.5	2.0	5.3	4.2
60	1.9	1.5	5.0	3.8	7.0	5.4
70	3.5	2.6	5.9	4.4	NA	NA

*NA* Not available

### Lung cancer epidemiology in India and Egypt

Further, we examined lung cancer incidence and mortality in India during the period 1980–2014 considering the age-standardized rate (Table 5) [2, 7, 35, 36]. The availability of these data from India was limited. The incidence of lung cancer for both men and women increased during 1980–2014.

**Table 5:** Age-adjusted rates of lung cancer incidence and mortality in India (1980–2014)

Year	Incidence (per 100,000 people)		Mortality (per 100,000 people)	
	Men	Women	Men	Women
1980	11.6	1.8	NA	NA
1985	11.2	1.3	NA	NA
1990	14.1	3.0	NA	NA
1995	13.0	3.7	NA	NA
2000	10.0	4.2	NA	NA
2005	10.7	5.0	NA	NA
2010	47.1	11.4	41.3	9.7
2011-2014	53.7	16.5	48.6	15.0

*NA* Not available

Finally, we examined lung cancer incidence and mortality in Egypt during the period 2000–2014. As in India, lung cancer incidence and mortality data were scarce in Egypt. Table 6 compiles the available data [2, 7, 37–39]. In Egypt, the mortality of lung cancer increased between 2000 and 2014; smoking was the main risk factor of lung cancer in Egyptians also [7, 37–39].

**Table 6:** Age-adjusted rates of lung cancer incidence and mortality in Egypt (2000–2014)

Year	Incidence (per 100,000 people)		Mortality (per 100,000 people)	
	Men	Women	Men	Women
2000	11.9	3.7	NA	NA
2005	14.0	3.0	NA	NA
2010	9.6	2.5	9.1	2.3
2011–2014	36.3	13.8	32.4	12.4

*NA* Not available

**Fig. 7:**
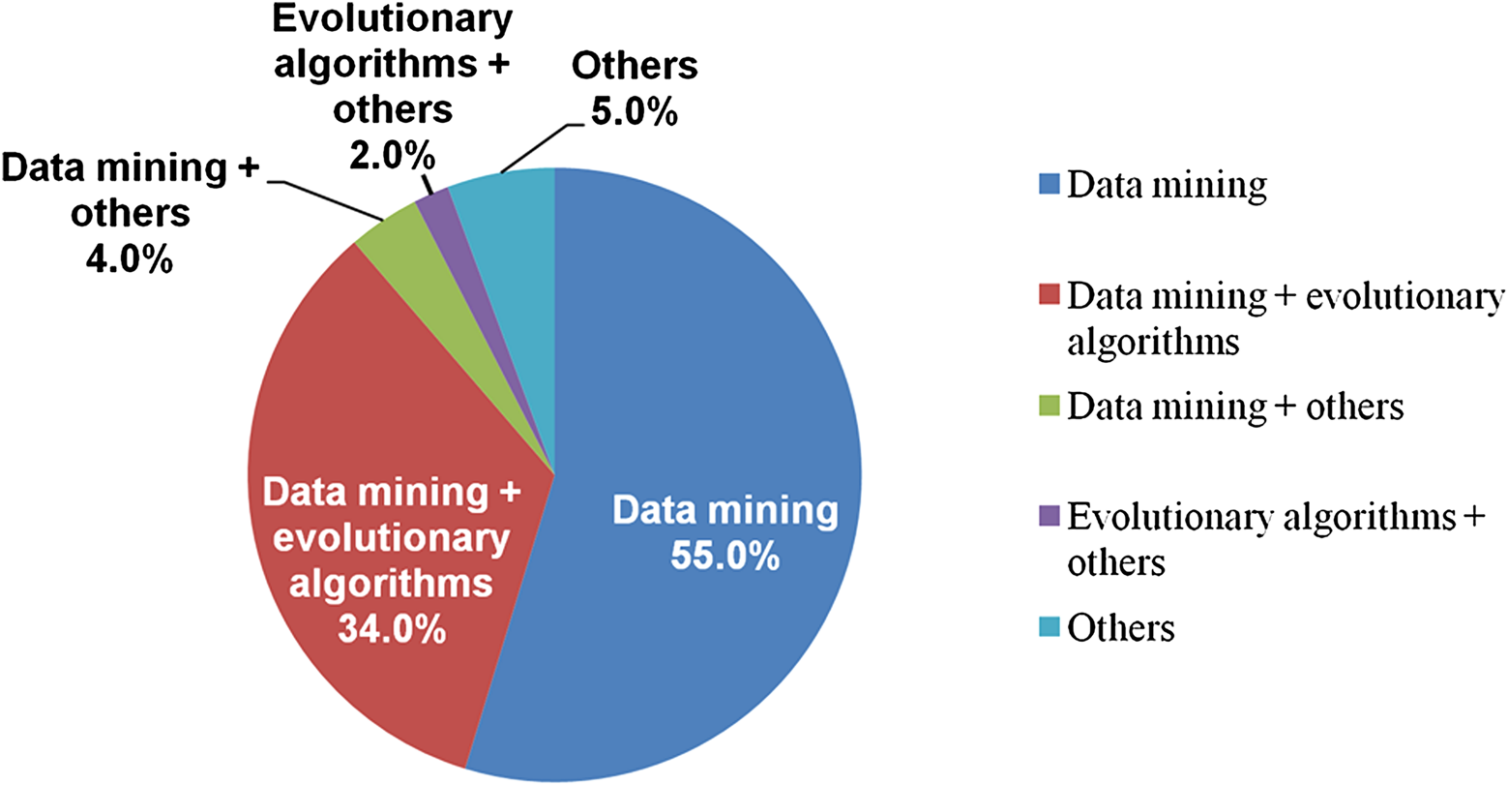
Percentage of different methods used in lung cancer diagnosis. It shows the frequency of data mining methods and evolutionary algorithms used for lung cancer diagnosis, as reported in references [43–70]

In the present study, we reviewed approximately 110 articles published by Elsevier, IEEE, and Springer during the period 2007–2015. We found that data mining and evolutionary algorithms were capable in efficiently classifying lung cancer data as depicted in Fig. 7. Previously, data mining methods were used alone by many researchers; however, our study indicated that the combination of data mining and evolutionary algorithms were more effective for the detection of lung cancer.

## Discussion

We found that currently the incidence and mortality patterns of lung cancer closely follow each other at the global level. In the US and the UK, advanced technology and awareness programs have helped decrease the mortality from lung cancer; however, this is not the case in India and Egypt, where more effective steps, such as development of special awareness programs, are required to decrease lung cancer mortality.

Cancer epidemiology is the study of causes and risk factors of a cancer for a given population. It can be helpful by allowing (i) the identification of health problems related to cancer, (ii) the measurement of the spread of the disease in a community, (iii) the expansion of knowledge about the risk factors of cancer, and (iv) a better understanding of the effects of cancer.

Cancer epidemiology can provide insights into the causes of cancer. However, the area under an epidemiologic investigation is often limited to a particular region and usually involves a small sample size. Since cancer epidemiology is analytical in nature, additional computational methods are required. Sample size can be increased easily, which can yield better classification results.

Data mining techniques can provide better classification and categorization of data, but these techniques may not efficiently cluster, classify, and predict the trends of sequential and time series data; hence, evolutionary algorithms are used to obtain optimal solutions in such cases. Evolutionary algorithms can produce high-quality analytical solutions and can simplify the problems during different iterative stages. Evolutionary algorithms such as ant colony optimization (ACO), particle swarm optimization (PSO), and artificial bee colony (ABC) are beneficial as these algorithms are capable of achieving a nearer solution in comparison to that achieved by the use of data mining techniques alone.

Pattern finding is very important in cancer detection. For this, data mining methods are needed. Data mining is a procedure by which pertinent patterns can be separated from large databases [40]. According to Jain et al. [41], data mining can be used for six specific tasks: classification, estimation, prediction, association rule mining, clustering, and visualization. Classification, estimation, and prediction are examples of supervised learning. The primary aim of these techniques is to prepare a model based on the available data, which can represent one or more attributes. Association rule mining, clustering, and visualization are examples of unsupervised learning. The primary aim of these techniques is to establish relationships between attributes. The six methods are used in nearly every area of healthcare databases for knowledge discovery, classification, and prediction. Of them, association rule mining, classification, and clustering are the most commonly used data mining techniques. These methods may provide a real solution for discovering similar types of groups, group patterns, the frequency of items present in the groups, the extraction of significant patterns, and pattern visualization [42]. Since lung cancer symptoms are not the same in every patient, it is essential to characterize their distinctive features and give unique treatments to different patients. In this regard, clustering or classification techniques may be useful because several factors, such as age, sex, genetics, alcohol consumption, smoking status, and weight may contribute to lung cancer.

According to Dass et al. [43], the two most important factors in cancer treatment are classification and characterization. They successfully achieved all the classification rules by using Apriori algorithm, which is helpful in the diagnosis of and the drug development for squamous cell cancer (SCC) and adenocarcinoma (ADC) [43]. Rajan et al. [44] suggested that the early diagnosis of lung cancer is mainly dependent on its historical data. Using association rule mining, Agrawal et al. [45] identified hotspots in lung cancer SEER data. A prototype mortality risk calculator was developed in this study; and the obtained rules satisfied biomedical knowledge. According to Yadav et al. [46], the detection of lung cancer is difficult at an early stage because it depends on multiple attributes. They used clustering approach for analyzing dataset from Sanjay Gandhi Post Graduate Institute of Medical Science, Lucknow, India. They compared the traditional clustering with foggy clustering method and achieved better results by using the latter. Piedra et al. [47] suggested text mining for a better understanding of the diagnostic process, classification accuracy, and disease facts; this can also be helpful in predictive model design, alert system, and decision-making process. Nahar et al. [48] used the association rule mining for identifying risk factors in different types of cancer. For this, they used three different types of association rule mining algorithms: the Apriori, predictive Apriori, and tertius algorithms. The Apriori algorithm outperforms the other algorithms. According to Wang et al. [49], Bayesian network is a very useful method for understanding cancer metastasis. The study included 50,000 cancer patients from Taiwan, China, between 1996 and 2010. Sensitivity and specificity measures were compared based on three different approaches, namely naive Bayes, logistic regression, and support vector machine (SVM), but the researchers did not find significant differences in terms of accuracy and specificity of the results. The interpretation capabilities of naive Bayes were superior to those of the other approaches, and it was also efficient in cases of missing information, modeling of non-linear situations, and stochastic medical problems. In their study, Krishnaiah et al. [50] examined decision tree, naive Bayes, and artificial neural network. One dependency-augmented naive Bayes classifier and one naive creedal classifier 2 were used for data pre-processing and decision making purposes, and the prediction results were better than the traditional methods. According to Phillips-Wren et al. [51], when decision tree and artificial neural network were used in combination, the chances of good prediction results became high. Debnath et al. [52] proposed a new evolutionary method for efficient classification of lung cancer genes. When a smaller number of genes were selected, this method provided better classification accuracy. According to Esfandiari et al. [53], data mining can determine the frequency of the task at a specified time. According to their study, data mining can be applied for disease prediction by data pre-processing and data modeling. Balachandran et al. [54] performed data mining to conduct a systematic study of lung cancer. The data were collected from medically confirmed and diagnosed patients. Their results showed that training-based approaches such as neural network performed better than cross-validation approaches. Fung et al. [55] proposed a new classifier that combined the impact factors (IFs) method and Golub and Slonim (GS) method with k-nearest neighbour (KNN). They achieved good classification performance for lung and prostate cancer data. Kushwah et al. [56] used neural network with random forest tree classifier for cancer gene selection. According to their results, classification capability could be increased with the help of trained neural networks. Guo et al. [57] used a network-based method on 164 smokers to identify the genes associated with smoking. They identified genes associated with lung cancer survival and genes that could distinguish smokers and non-smokers; the accuracy of the method was 73.0%. Ahmed et al. [58] prepared a database of 400 patients that comprised patients with or without cancer. For pre-processing, k-means clustering was used. The results proved that this method was efficient in lung cancer risk identification. Sun et al. [59] suggested that the SVM can be used for lung cancer classification, based on the comparison of different algorithms, such as boosting, decision tree, and KNN. Oztekin et al. [60] proposed a prediction model based on decision tree, neural network, and logistic regression. The study suggested that these algorithms were capable of accurate classification of the lung cancer dataset.

Evolutionary algorithms are population-based meta-heuristic optimization algorithms that are inspired by nature. The principal evolutionary algorithms are genetic algorithm (GA), ACO, PSO, ABC, and memetic algorithm. Now, evolutionary algorithms, in combination with the previously discussed methodologies, are being discussed. These algorithms can locate the closest solution even when dealing with complex issues.

Li et al. [61] proposed a bionic enhancement calculation-based system, termed ant colony optimization-selection (ACO-S) for high-dimensional datasets. The outcomes demonstrated that ACO-S could produce a high-quality subset with a small size and better characterization. Yu et al. [62] recommended ACO sampling to address the issue of class unevenness. The methodology resulted in greater grounded speculation capacity as compared with the traditional methods. Sowmiya et al. [63] suggested neural network and fuzzy logic to train data. Then, by using ACO, classification accuracy was improved. Alba et al. [64] compared PSO and GA. They used SVM in combination with either of the algorithms on high-dimensional microarray data for classification. The combination of PSO and SVM was capable of finding interesting genes. Minimum redundancy maximum relevance (MRMR)-GA was compared with GA-SVM wrapper and MRMR filter. In terms of selection and classification performance, MRMR-GA produced better results. Qasem et al. [65] presented a new multi-objective algorithm based on swarm optimization for classification problems, termed multi-objective particle swarm optimization RBF network (MPSON). The results indicated that this method had good generalization capability along with compact network structure. Runkler et al. [66] made efforts to minimize fuzzy c-means model using ACO, alternate optimization (AO), and PSO. They suggested two different forms of PSO: the first was PSO–V for representing particle as a component of a cluster center; the second was PSO–U for representing particle as a non-scaled and non-normalized membership value. PSO–V and PSO–U were compared with AO and ACO. They were compared with two different datasets: single outlier and lung cancer. The results of ACO, PSO–V, and PSO–U were slower than AO, but PSO variants outperformed significantly after each round of iteration. Liu et al. [67] suggested discrete particle swarm optimization (DPSO) and rule pruning for lung cancer diagnosis and achieved 68.3% classification accuracy. Liu et al. [68] used the PSO-based simultaneous learning framework for clustering and classification (PSOSLCC). PSOSLCC was applied to a real-world application, namely texture image segmentation, and good performance was obtained, showing that it could potentially classify problems on a large scale. Chen et al. [69] proposed an approach based on PSO with a decision tree classifier for statistical analysis. They found that this method outperformed other popular classifiers (i.e., SVM, self-organizing map, back propagation neural network, and C4.5 decision tree) by conducting experiments on 11 gene expression cancer datasets. Subbulakshmi et al. [70] proposed an efficient hybrid approach based on PSO with an extreme learning machine classifier. It had self-regulated learning capability that showed good generalization performance. These studies above suggested that the data mining and evolutionary algorithms both are efficient in lung cancer detection; while the evolutionary algorithms have the capabilities of handling complex problems, the data mining algorithms alone may fail. Therefore, combining both approaches at different levels of classification and clustering may produce better outcomes.

**Fig. 8:**
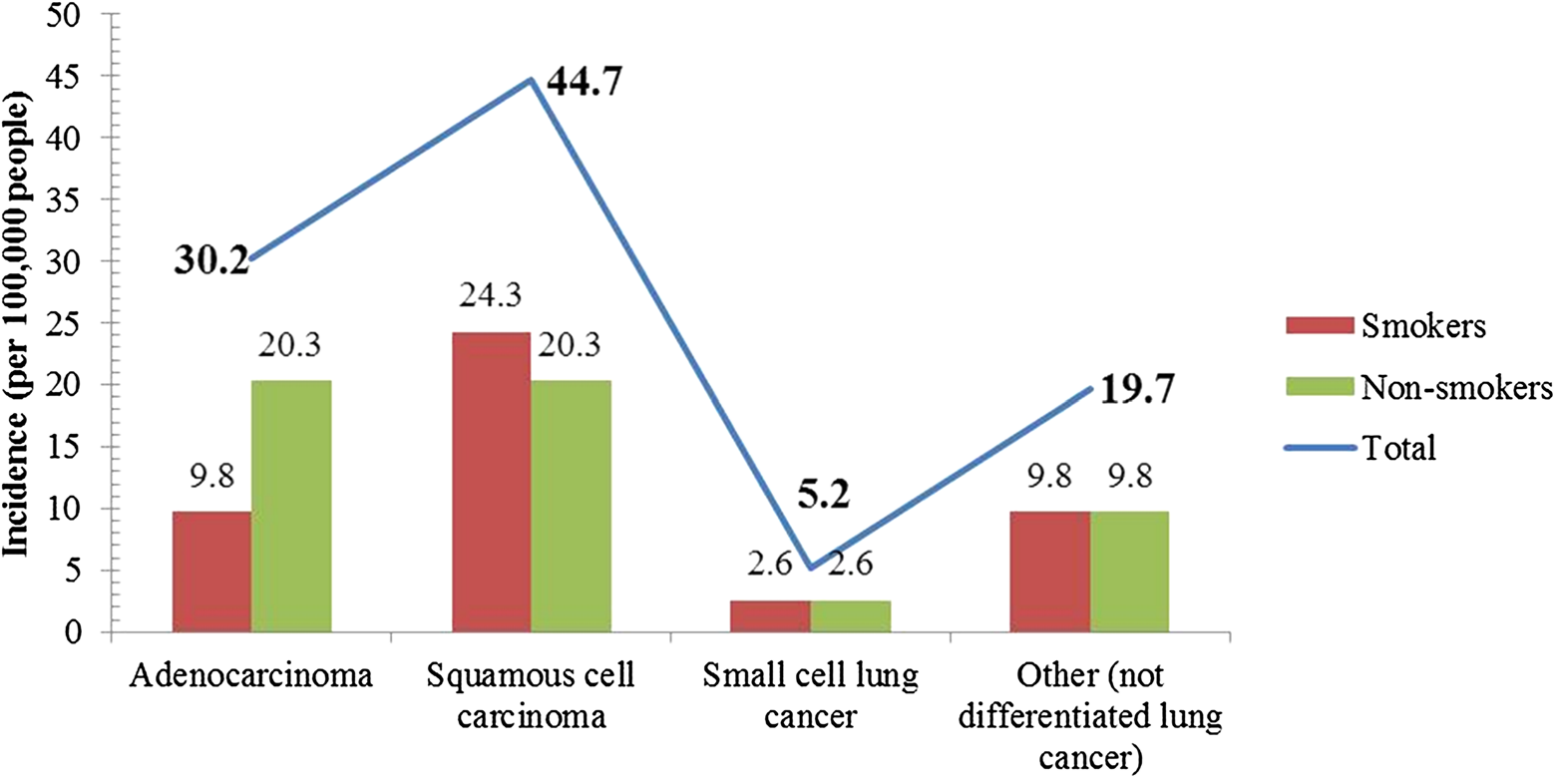
Relationship of the incidence of various histological types of lung cancer with smoking in India

The use of tobacco products causes approximately 5 million deaths worldwide annually, with 2.41 million deaths in developing countries and 2.43 million deaths in developed countries [71–73]. Of the 5 million deaths that occurred annually in India, approximately 1 million could be attributed to cancer [71, 74]; by 2020, this figure is estimated to reach 1.5 million [40]. Smoking is responsible for 80.0% of the lung cancer incidences worldwide [71–74]. In India, cigarette or beedi smoking causes the majority of the deaths in the 25–69 age group [71, 75]. Some studies have reported that 15.0% of lung cancer cases were caused by genetic factors, air pollution, or exposure to radon gas, asbestos, and pesticides [71, 76, 77]. These studies also showed that, Indian non-smokers have almost the same chance of getting lung cancer as smokers because of exposure to pesticides and other carcinogens (Fig. 8) [36, 77]. Our results suggest that there must be strict restrictions on the use of tobacco products.

### Study limitations and future directions

This study has some limitations. Firstly, maximum collection of data was based on the continuous availability of data, but in some cases the data were from one-time community- or hospital-based surveys. The incidence and mortality data from India and Egypt may not be complete. Therefore, deviations in incidence and mortality from the actual are possible. However, these errors may be negligible, since as much data as possible were taken from identified sources and published papers. Secondly, we considered lung cancer statistics only from the UK, the US, India, and Egypt. The results will vary if more countries are considered. Thirdly, further research is required to clarify how data mining and evolutionary algorithms can be used together, and which combined techniques will be most effective. Finally, only English-language sources and publications were examined.

## Conclusions

In developed countries, such as the UK and the US, lung cancer mortality is declining and a high survival rate has been achieved, likely due to awareness programs and advanced medical technologies. However, in developing countries such as India and Egypt, substantial efforts are need to decrease cancer mortality.

We also analyzed computational methodologies for their usefulness in the early detection of lung cancer. It was found that data mining techniques such as classification, clustering, and association rule mining were most commonly used but a better outcome could be achieved if data mining is combined with the evolutionary algorithms. We also found that when lung cancer symptoms were identified correctly, the chances of detection increased; and for this classification, clustering techniques of data mining could be employed. The chances of getting good results are lower with a single method, since the characteristics of lung cancer may be different. Data mining along with evolutionary algorithms can better characterize lung cancer symptoms at different levels, arrange them in groups, and determine rankings to allow their stage and behavior being identified correctly and timely.
